# Utilization of Gelling Polymer to Formulate Nanoparticles Loaded with Epalrestat-Cyclodextrin Inclusion Complex: Formulation, Characterization, In-Silico Modelling and In-Vivo Toxicity Evaluation

**DOI:** 10.3390/polym13244350

**Published:** 2021-12-12

**Authors:** Zunaira Alvi, Muhammad Akhtar, Nisar U. Rahman, Khaled M. Hosny, Amal M. Sindi, Barkat A. Khan, Imran Nazir, Hadia Sadaquat

**Affiliations:** 1 Department of Pharmaceutics, Faculty of Pharmacy, The Islamia University of Bahawalpur, Bahawalpur 63100, Punjab, Pakistan; zunairaimran3988@gmail.com (Z.A.); hadiasdqt@gmail.com (H.S.); 2 Department of Medical Laboratory Technology, Faculty of Medicine and Allied Health Sciences, The Islamia University of Bahawalpur, Bahawalpur 63100, Punjab, Pakistan; 3 Department of Pharmacy, Royal Institute of Medical Sciences (RIMS), Multan 60000, Punjab, Pakistan; nisar60@yahoo.com; 4 Department of Pharmaceutics, Faculty of Pharmacy, King Abdulaziz University, Jeddah 21589, Saudi Arabia; kmhomar@kau.edu.sa; 5 Department of Oral Diagnostic Sciences, Faculty of Dentistry, King Abdulaziz University, Jeddah 21589, Saudi Arabia; amsindi@kau.edu.sa; 6 Drug Design and Cosmetics Lab (DDCL), Gomal Center of Pharmaceutical Sciences, Faculty of Pharmacy, Gomal University, Dera Ismail Khan 29050, Khyber Pakhtoonkhwa, Pakistan; barkat.khan@gu.edu.pk; 7 Bahawal Victoria Hospital, Bahawalpur 63100, Punjab, Pakistan; chimransms@gmail.com

**Keywords:** epalrestat, inclusion complex, solubility, chitosan nanoparticles, in-silico modeling, in-vivo toxicity evaluation

## Abstract

Epalrestat (EPL) is an aldose reductase inhibitor with poor aqueous solubility that affects its therapeutic efficacy. The research study was designed to prepare epalrestat-cyclodextrins (EPL-CDs) inclusion complexes to enhance the aqueous solubility by using beta-cyclodextrin (β-CD) and sulfobutyl ether₇ β-CD (SBE_7_ β-CD). Furthermore, polymeric nanoparticles (PNPs) of EPL-CDs were developed using chitosan (CS) and sodium tripolyphosphate (sTPP). The EPL-CDs complexed formulations were then loaded into chitosan nanoparticles (CS NPs) and further characterized for different physico-chemical properties, thermal stability, drug-excipient compatibility and acute oral toxicity studies. In-silico molecular docking of cross-linker with SBE_7_ β-CD was also carried out to determine the binding site of the CDs with the cross-linker. The sizes of the prepared NPs were laid in the range of 241.5–348.4 nm, with polydispersity index (PDI) ranging from 0.302–0.578. The surface morphology of the NPs was found to be non-porous, smooth, and spherical. The cumulative percentage of drug release from EPL-CDs loaded CS NPs was found to be higher (75–88%) than that of the pure drug (25%). Acute oral toxicity on animal models showed a biochemical, histological profile with no harmful impact at the cellular level. It is concluded that epalrestat-cyclodextrin chitosan nanoparticles (EPL-CDs-CS NPs) with improved solubility are safe for oral administration since no toxicity was reported on vital organs in rabbits.

## 1. Introduction

Epalrestat (EPL) is relatively a new class of drug, prescribed in Japan as an anti-diabetic drug. Its primary mode of action is the suppression of aldose reductase (AR), an enzyme involved in the polyol pathway. In the presence of NADH, AR transforms glucose into fructose, which is later converted into sorbitol. Increased AR expression has been linked to diabetes mellitus since it makes tissues depend on insulin for the absorption of glucose. When provided at the recommended dose of 150 mg/day, EPL decreases oxidative stress in Type-II diabetes by lowering lipid peroxidase levels in RBCs [1]. Unfortunately, EPL has limited solubility in water and has been shown to have dissolution problems. Different approaches have been used in recent years for increasing the solubility and bioavailability of hydrophobic drugs. Inclusion complexation is one such approach that favorably enhances the solubility of lipophilic drugs by forming a reversible correlation between the ligand (EPL) and the substrate (CDs) [2,3,4]. The pharma industries are also taking a keen interest in the production of NPs because they have the ability to improve the solubility of poorly soluble drug substances by introducing nano-sized drugs in systemic circulation [5]. NPs are actually solid colloidal particles that may be either nanocapsules or nanospheres [6] and can be prepared by different methods. In this research study, NPs were prepared through the ionotropic gelation method using cationic polymers such as CS. The CS is a carbohydrate polymer synthesized from chitin by partial N-deacetylation. The prime component in the chitin is the acetyl amino group bound to β-(1,4) glucosidic linkage creating the repeating units of linear polymers. CS is available in a variety of molecular weights. The degree of deacetylation and weight are the significant parameters that influence the size of the particle [7]. Conversely, while working with lipophilic drugs, the cationic nature of CS impedes its application. Recently chitosan-cyclodextrin (CS-CD) nanocarrier systems have been fabricated. This innovative approach can successfully incorporate the lipophilic drug by creating the weak host-guest association termed as “Inclusion Complexation”. This phenomenon of complexation increases the physical and chemical properties of drugs, consequently increasing the solubility and stability, decreasing the drug toxicity, regulating the drug release and enhancing the drug performance [8]. The benefits of implementing this technique are combining the promising behavior of the CS nanoparticles (CS NPs) with the improved biopharmaceutical qualities of the CDs. In fact, CDs are very well recognized in the pharmaceutical industry owing to their ability to protect the drug from certain environmental conditions and, therefore, strengthen its membrane permeability.

β-CD is an acyclic oligosaccharide containing 7 α-D glucopyranose complex [9,10]. The size of the cavity is an important parameter for the incorporation of the drug into the cavity. Only those drugs with a molecular weight of less than 800 mg/mol are suitable candidates for this host-guest association [11]. Besides this natural CD, different chemical modifications in the CD have been manufactured and new derivatives developed [12]. The sulfobutyl substituted CD is among the most popular groups of modified CDs. Sulfobutyl ether is the derivative of polyanionic CD that has better aqueous solubility compared to β-CD [13]. Though natural CDs demonstrated poor solubility in water compared to their derivatives, even then the solubility was enough to cope with the problems of drug absorption at the target site [14]. These unique attributes of β-CD and SBE_7_ β-CD make them suitable candidates for the synthesis of polymeric nanoparticles (PNPs) using the ionotropic gelation method.

The aim of the designed study was to formulate EPL-CDs complexed CS nanoparticles by using chitosan cross-linked with sodium tripolyphosphate through the ionotropic gelation method. Out of the six prepared nanoparticle formulations, one was taken as optimized based on the particle size analysis and in-vitro drug release study and thereby further evaluated for oral toxicity studies, using rabbit as an animal model.

## 2. Experimental Details

### 2.1. Chemicals and Reagents

The following chemicals and reagents have been purchased from commercial sources and were used as received. Epalrestat (CAS No. 82159-09-9), kindly gifted by Symed labs, Hyderabad, India, β-CD (CAS No. 7585-39-9, MW = 1135 Da, Sigma-Aldrich, Steinheim, Germany), SBE_7_ β-CD (CAS No.182410-00-9, MW = 2160 Da, average degree of substitution = 6.50, Cydex, Lenexa, KS, USA), chitosan hydrochloride (CAS No. 70694-72-3, MW = 110 KDa, deacetylation degree = 85% according to the manufacturer specifications, Sigma-Aldrich, Steinheim, Germany), sodium tripolyphosphate (CAS No. 70694-72-3, Sigma-Aldrich, Taufkirchen, Germany), glacial acetic acid (purity 99%, BDH, Laboratory supplies, UK), Tween 80 (Merck, KGa, Darmastadt, Germany), ethanol (Sigma–Aldrich Chemie, Steinheim, Germany), hydrochloric acid (37% BD, Laboratories supplies, UK), potassium dihydrogen phosphate (MP, Biomedicals, China), sodium hydrogen phosphate (Merck, Darmastadt, Germany), acetone (Sigma-Aldrich, Steinheim, Germany) and dialysis tubing membrane (having cut-off value 12–14 K Da). The CDs were kept in a desiccator to protect them from moisture until further use. Ultra-pure water (Millipore, Billerica, MA, USA) was used in the study in order to prepare certain aqueous solutions.

### 2.2. Methodology

#### 2.2.1. Preparation of Binary System

##### Physical Mixing Method (PM)

Physical mixture (PM) of EPL with β-CD and SBE₇ β-CD was synthesized by triturating drug and CDs (1:1) separately in pestle and mortar for about 60 min [15]. The prepared mixtures were sieved through mesh no. 45 and stored in a tightly closed vial.

##### Kneading Method (KM)

Adequate quantities of EPL and β-CD (1:1) were weighed accurately. The homogenous solution of EPL was prepared by dispersing EPL in ethanol. A uniform paste of β-CD was prepared in pestle and mortar, using water. The solution of EPL was added to this homogenous paste in a portion and continuously kneaded for about 60 min. The mixture was dried in an oven (FTC 90 E Refrigerated incubator VELP SCIENTIFICA, Europe) at 45–50 °C for 24 h. Finally, the dried complexes were lyophilized (lyophillizer, Christ Alpha 1-4 LD, Germany) and stored in an airtight container [16]. The above-mentioned procedure was repeated to obtain the complexes of EPL with SBE_7_ β-CD.

##### Co-Precipitation Method (CP)

Equal quantities of EPL and β-CD were weighed accurately. The drug was dissolved in 10 mL of acetone, while the measured quantity of β-CD was dispersed in 5 mL of distilled water previously warmed to 75 ± 0.5 °C. To this β-CD solution, a solution of EPL dropwise with temperature maintenance of 75 °C was added. The mixture was allowed to stir for about 60 min, left to cool down to 25 °C and again stirred (heating magnetic stirrer, ARE VELP SCIENTIFICA, Europe) for 30 min. EPL precipitated out from solution. Dried the mixture at 45–50 °C in hot air oven, (FTC 90 E Refrigerated incubator VELP SCIENTIFICA, Europe) overnight. Lyophilized (lyophilizer, Christ Alpha 1–4 LD, Germany) the dried complex and repeated the same procedure for complexation of EPL with SBE_7_ β-CD [17,18].

#### 2.2.2. Preparation of Drug Unloaded CS/sTPP NPs

Blank NPs were prepared by the method adopted by [19] with slight changes. The optimized procedure was designed by changing different parameters like concentration of CS and acetic acid (AA), volume of sTPP, pH of the solution and ratio of CS/sTPP [20] as given in Table 1. For all the test samples, the temperature and the stirring speed of the reaction mixture remained constant. The impact of these parameters was therefore studied and the criteria for the selection of the best formulation was based on particle size analysis.

Test sample no. 5 was reported to be the optimized test sample and the procedure adopted for the preparation of NPs was as follows: For the preparation of CS solution, low molecular weight (LMW) CS (0.1%) was dissolved in AA solution (2% *v/v*). The prepared solution was allowed to stir at 500–600 rpm for 4 h to obtain the homogenous mixture. The pH of the resultant mixture was 3.7 and adjusted at 4.6 using NaOH solution (0.1N). The solution was filtered using syringe filters with 0.45 μm pore size (Millipore, USA). To this solution, 1 mL of sTPP solution (cross-linker) dropwise was added with constant stirring. After about 1 h the solution became opalescence, which is a clear indication for the formation of NPs.

#### 2.2.3. Preparation of EPL-CDs Loaded CS NPs

EPL-CDs loaded CS NPs were synthesized in two steps. Firstly, EPL was complexed with β-CD and SBE_7_ β-CD using physical mixing (PM), kneading method (KM) and co-precipitation (CP) method, and was further fabricated into NPs by following the procedure given below.

A total of 10 mg/mL of aqueous solution of both the complexes were added dropwise in 6 mL of CS solution and stirred (VELP Scientifica, Usmate, Italy) for about half an hour to obtain the clear solution. The molar stoichiometric ratio selected for EPL and the CDs was 1:1. NPs were abruptly generated by adding 1 mL (1% *w/v*) of sTPP solution under magnetic stirring over the period of 60 min at the speed of 700 rpm. At this stage, 17 µl of tween 80 was added to enhance the stability of the prepared NPs. The solution was centrifuged (Sigma-Zentrifugen, Osterode, Germany) for approximately 4 min at 12,000 rpm [21]. The supernatant was subjected to determine the entrapment efficiency through indirect method. The solution was slurred in water to redisperse it. Finally, the mixture was vortexed (MS2 minishaker IKA, Osterode, Germany) and lyophilized (Martin Christ Alpha-1-4LD freeze-drier, Osterode, Germany) to get NPs that were coded as NP1, NP2, NP3, NP4, NP5 and NP6. The obtained NPs were stored in airtight, light resistant container at 4 °C. Table 2 depicts the coded formulations prepared by different methods and complexes.

### 2.3. Characterization of EPL-CDs-CS NPs 

#### 2.3.1. Particle Size, Zeta Potential (ζ) and Polydispersity Index (PDI)

Size of the polymeric NPs is a very important parameter; NPs should be small enough to cross the semi-permeable biological membranes and allow particles sized 200 nm to escape the reticulo-endothelial junctions. The prepared NPs should have the homogeneity for their effective performance [22], therefore, the NPs with zeta potential in the range of 20–40 mV had the sufficient repulsive force to remain in an aqueous dispersion [23]. For drug unloaded NPs, the test samples prepared using different parameters were analyzed by DLS (Malvern Instruments, Malvern, UK). The prepared NPs were diluted using phosphate buffer pH 6.8 for analysis. Disposable cuvettes were used for size determination. For drug unloaded NPs, test sample no.5 was selected as the best formulation and was evaluated further to get the best results [24]. Similarly, the same procedure was adopted for the drug-loaded NPs, and particle size analysis was carried out.

#### 2.3.2. Yield (% *w*/*w*)

Yield (% *w*/*w*) of the prepared NPs was calculated as weight (mg) of the dried NPs from each batch to sum of initial weight (mg) of EPL, CS and cross-linker multiplied by 100, as shown in Equation (1).

(1) $$\mathrm{Yield}\;\left(\%\frac{w}{w}\right)=\frac{\mathrm{wt}\;\mathrm{of}\;\mathrm{dried}\;\mathrm{Nps}\;\mathrm{recovered}\;\left(\mathrm{mg}\right)}{\mathrm{wt}\;\left(\mathrm{mg}\right)\mathrm{of}\;\mathrm{EPL}+\mathrm{wt}\;\left(\mathrm{mg}\right)\mathrm{of}\;\mathrm{CS}+\mathrm{wt}\;\left(\mathrm{mg}\right)\mathrm{of}\;\mathrm{sTPP}}\times 100$$

#### 2.3.3. EPL Entrapment Efficiency in NPs

The amount of EPL entrapped in nanoparticulate system was calculated through indirect method. The measurement for quantifying the amount of EPL (mg) entrapped in the system was carried out using a UV-VIS spectrophotometer (IRMECO GambH, Geesthact, Germany) at λ = 388 nm. The formula used for measuring the % EE for loaded NPs is given in Equation (2).

(2) $$\mathrm{EE}\;\left(\%\right)=\frac{\mathrm{Total}\;\mathrm{EPL}-\mathrm{Free}\;\mathrm{EPL}\;}{\mathrm{Total}\;\mathrm{EPL}}\times 100$$

#### 2.3.4. Fourier Transform Infrared Spectroscopy (FT-IR) 

To determine the compatibility between EPL, CDs polymer, cross-linker, and optimized formulation (NP1, NP2, NP3, NP4, NP5 and NP6), an FT-IR analyzer (α-Bruker, tensor, 27 Series, Karlsruhe, Germany) was used. FT-IR works on the ATR principle in which infrared rays emitted are absorbed by different chemical bonds and functional groups in a particular range of 4000–500 cm^−1^. Samples in lyophilized form were ground and placed on ATR crystal and force is applied by using plunger by rotating and turning arm so that it may be pressed tightly. Finally, the spectral data were analyzed using OPUS software.

#### 2.3.5. Powder X-ray Diffraction Pattern (PXRD)

Powder X-ray diffractometry (D-8 Advance α-Bruker, Karlsruhe, Germany) was used to examine the trend of diffraction of EPL, CDs, CS and sTPP and the formulations were assessed under operating condition of 40 mA and voltage of 40 kV. The origin used for generating X-ray radiations were Cuα. The specimens were examined at a rate of 2θ/min in the range of 20–50°. The obtained results were analyzed and compared for the presence of peaks, their position, and their shifting.

#### 2.3.6. In-Vitro Studies

In-vitro drug release studies of EPL and NPs (NP1, NP2, NP3, NP4, NP5 and NP6) was studied using modified dialysis bag technique. This is a glass apparatus with 100 mL of buffer (pH 6.8) as the release media at 37 °C. Weighed quantity of pure EPL and NPs were placed in a dialysis bag, which was previously soaked for 12 h in release media. The open ends of the dialysis membrane were secured with two clamps. The system was tightly closed to keep the medium from evaporating. The sample of 3 mL was then withdrawn at 0, 15, 30, 45, 60, 90, 120, 180, 240, 360, 480 and 720 min by maintaining sink condition. Each sample withdrawn was filtered using syringe filters. The concentration of EPL in NPs was analyzed using UV-VIS spectrophotometer [13] at 388 nm wavelength. The NPs, which are best in characterization (EE, Yield and in-vitro drug release), were selected as optimized formulations.

#### 2.3.7. Drug Release Kinetics

The drug release from the NPs can be carried out through the process of leaching. The release media enter the polymer matrices through pores, cavities, cracks and spaces. Drug release from matrix involves diffusion, erosion and dissolution [25]. In-vitro dissolution study is an important parameter in drug development. The nature of the drug, its morphology, crystallinity, size, aqueous solubility, and its percentage in dosage form can, in turn, affect the kinetics. Depending on the correlation coefficient (r), the mathematical model that suited best to release data was selected. The model with the highest value of “r” is the best in terms of drug release.

##### Zero-Order Kinetics

The slow drug release from the polymer and no disintegration are better fitted in zero-order kinetics. This system of kinetics described that the rate of drug release is independent of the concentration of drug. The formula for zero-order kinetics is given in Equation (3).

(3) $$Q_{t}=Q_{0}+k_{0}t$$

$Q_{t}$ = Total amount of drug release at time *t*

$Q_{0}$ = Amount of drug at time *t* = 0

$k_{0}$ = Release constant

The graph plotted between drug release against time will be linear for zero-order kinetics.

##### First Order Kinetics

The first-order kinetics described that the drug release from formulation depends upon the drug concentration Mathematically, it is expressed in Equation (4) [26].

(4) $$logC_{t}=logC_{0}-\frac{kt}{2.303}$$

$C_{t}$ = Concentration of drug at time *t*

$C_{0}{}_{}$ = Concentration of drug at time *t* = 0

k = rate constant

##### Higuchi Model of Kinetics 

The release of drug was defined as a diffusion-controlled mechanism based on Fickian law. The planar system with homogeneity is described in Equation (5) [27].

(5) $$Q=k_{h}t^{1/2}$$

Q=Cumulative drug release at time t

$$k_{h}=Higuchi\;constant$$

##### Korsmeyer-Peppas Release Kinetics

In 1984, Korsmeyer and Peppas devised an empirical equation to describe Fickian and non-Fickian drug release from swellable and non-swellable polymer systems. The formula used by Korsmeyer-Peppas is given in Equation (6) [27].

(6) $$\frac{M_{t}}{M_{\alpha }}=kt^{n}$$

#### 2.3.8. In-Silico Molecular Docking

In-silico modeling is computer-based docking that is aimed to estimate the non-covalent interaction of receptor (macromolecule) with ligand (small molecules). The prediction of binding the receptor with ligand is very important since it is used to evaluate further drug development processes. Therefore, in-silico molecular modeling was conducted to determine the interacting site of SBE_7_ β-CD with cross-linker (TPP) by using AutoDock-1.5.6, vina and Biovia/Discovery studio 2021. The 3D structure of SBE_7_ β-CD was not available, therefore, the structures were drawn in Chem3D pro12.0. The file was then converted to PDB (protein data bank) format using OpenBabel-2.4.1. AutoDock tool was used to process the files of ligand and receptor. Finally, for visualization of the obtained results, Biovia//Discovery studio was used.

#### 2.3.9. Morphology of Prepared NPs

SEM technique was employed to determine the morphology and the appearance of the NPs. The lyophilized powder of the prepared NPs was placed on an aluminum specimen covered with a carbon adhesive disc and sputter with gold. After coating, the images were captured at high-resolution [28]. 

The surface of the NP was assessed using (TEM). For this purpose, the voltage of (TEM) was set to 200 kV. One drop of diluted sample was spread on the copper grid coated with carbon, allowing the sample to dry at room temperature before inspection at suitable magnification [29].

#### 2.3.10. Differential Scanning Calorimetry (DSC)

DSC of optimized formulation (NP6) was carried out by placing 5 mg of lyophilized powder on aluminum pan heated in the range of 20–200 °C at a flow rate of 30 mL/min. Indium and zinc were utilized as standards, while nitrogen was employed as a purge gas.

#### 2.3.11. Toxicity Studies

The toxicity studies of CS cross-linked sTPP NPs were tested on albino rabbits, as per regulations of OECD (Organization for Economic Co-operation and Development) [30]. The permission for this research study was given by the “Pharmacy Animal Ethics Committee” (PAEC), Faculty of Pharmacy, The Islamia University of Bahawalpur, having reference no. 21/2020/PAEC, dated 17 September 2020. The purpose for conducting the toxicity studies of nano-system was to examine the toxic effects produced by the system and its distribution throughout the body [31]. Albino rabbits were considered for study because of the data available and the established pathophysiology that can anticipate consequences on human health. A total of 12 healthy albino rabbits were carefully selected and weighed and categorized in two groups of six rabbits each. The animals selected were not a part of any research study. The living conditions for animals were in accordance with the guidelines i.e., 20 ± 3 °C temperature, humidity less than 40% and artificial light with light and dark sequences. The animals were placed in separate clean wooden cages with access to food and water. Each group was labeled as Group A for control and Group B for test formulations. All the animals in Group A were given only water, and the animals in Group B were given test formulation. Throughout the testing period, the record of toxicity, weight and food consumption were observed [32,33]. The rabbits were slaughtered. Their organs were removed and stored in 10% buffered formaldehyde solution. Blood samples were withdrawn and preserved in EDTA (ethylenediaminetetraacetic acid) tubes for hematological and histopathological studies [34].

## 3. Results and Discussion

### 3.1. Formation Mechanism for CS/sTPP NPs

CS by inter and intramolecular H-bonding has a rigid crystalline structure. In the aqueous phase, due to repulsive forces between the chain system, CS developed the modified orientation with a somewhat stable but flexible chain. NPs are formed after CS and sTPP are combined in acetic acid (diluted) solution, mostly as a consequence of the relationship between phosphate ions of sTPP (negatively charged) and the amino group of CS (positive charge). Additionally, because of the protonated amino groups of CS, there have been repulsive forces between the CS and interchain H-bonds around the CS molecules [35].

### 3.2. Evaluating the Condition for the Optimization of NPs

Numerous physico-chemical and mechanical parameters can influence the specific interaction between the CS and sTPP. Initially, different parameters were optimized by changing the concentration of CS, the volume of sTPP, concentration of AA, pH of the solution and the ratio of CS/sTPP for the successful fabrication of NPs in Table 3.

#### 3.2.1. Effect of Concentration of CS

The behavior of developed CS/sTPP NPs with different concentration of CS has been investigated and depicted in Table 3. The findings indicate that when the concentration of CS increases, so does the size of the NPs. According to [19], a particular concentration of CS was preferable for the synthesis of NPs. This important aspect has been checked in our study and it was found that the concentration of CS must have been in a range between 0.1–0.2% (1–2 mg/mL) in order to prevent any microparticles. The interchain H-bonding and the repulsive forces because of the protonated amine groups are developed within the acceptable range (1–2 mg/mL). These attractive and repulsive forces are indeed in equilibrium. However, when the concentration of CS rises, the molecules of CS come close with a restriction across this concentration range, resulting in a slight increase in electrostatic attraction and thereby generating bigger, yet nano-sized particles. When the concentration increases, microparticles are formed because of the H-bonding. At the same time, the repulsive forces between the particles start diminishing and are not adequate to maintain the equilibrium. These developed particles normally lead to precipitation, which can be clearly seen at the bottom of the vessel [36].

#### 3.2.2. Effect of Volume of sTPP

The possible cross-linking site present on the structure of CS is because of the degree of acetylation and protonation, which imparts a positive charge onto the CS molecule. Table 3 described the relevancy of CS on the particle size by changing the volume of sTPP. It can be inferred from the reported results that the size of the NPs dramatically increased by increasing the volume of sTPP from 1–1.5 mL. Initially, when the volume of sTPP is below 1 mL, the reaction mixture is transparent without noticeable opalescence, which implies that the volume of sTPP was insufficient to aid in the development of cross-linked CS. But when the volume of sTPP is further increased to 1.5 mL, the molecules of CS are completely cross-linked and, as a result, the additional sTPP will be contributed to the development of large-sized NPs having more CS molecules.

#### 3.2.3. Effect of Concentration of AA

The concentration of AA reported to disperse CS is either 1% or 2%. If the concentration is further increased, then the volume of NaOH can also increase in order to neutralize the acid and change the pH of the CS solution, as the pH is an important parameter for the fabrication of CS/sTPP NPs. The increased concentration of AA induced the change in ionic strength, which, in turn, enhanced the shielding effect of CH_3_COO^−1^ counter ions, ensuring that CS molecules have a limited cross-linking site for sTPP. The enhanced shielding effect declined the repulsive force within the particles and, therefore, promoted the accumulation of the particles with the larger size, resulting in a large proportion of microparticles [37].

#### 3.2.4. Effect of pH on CS Solution

For CS molecules, the degree of protonation is primarily affected by the pH of the CS solution, as CS is an aqueous soluble polyelectrolyte with a pKa of 6.5. The relation between the pH of CS and protonation has already been discussed [38]. The findings suggested that as the pH of the solution increased to above 7.0, the degree of protonation for CS fell from 100% to 0%. From the results, it can also be inferred that there is a range of pH above which the protonated structure of CS starts deprotonating [20]. For this purpose, the pH effect on the size of NPs was investigated within the range of 3.6–5.6. Table 3 depicted the results of the study. From the results, it can be assessed that when the pH of the solution is below 4.6, NPs are not formed as the solution is clear and no opalescence solution can be seen. When the pH is above 4.6, the presence of precipitates indicates that microparticles are present in the solution. Therefore, pH 4.6 has been selected as the suitable pH for the synthesis of NPs.

#### 3.2.5. Optimization of CS/sTPP Ratio

The crucial parameter for the synthesis of NPs has been reported to be the CS/sTPP mass ratio, as it affects the sufficiency of both CS and sTPP. The role of the CS/sTPP ratio on the development of NPs has been reported in Table 3 by altering the ratio 5:1, 6:1 and 7:1. In the study, the concentration of CS is increased while the volume of sTPP is kept constant. From the obtained results, it can be assessed that by increasing the ratio of CS/sTPP, the size of NPs increases from 510 to 1048 nm. It can also be inferred that low concentration of CS synthesized stable NPs at a low ratio of CS/sTPP. When the concentration of sTPP is 0.1% *w/v*, and the concentration of CS is 0.1% *w/v*, the stable NPs can be developed at the ratio of 6:1. However, when the concentration of CS is 0.2% *w/v* and sTPP is 0.1% *w/v*, the NPs come close to each other and form the flocculation at ratio 7:1. This justifies the effect of the distance between the molecules increasing as the concentration of CS decreases, thereby affecting the cross-linking sites of CS molecules. In our study, 6:1 is the optimized ratio for the successful fabrication of NPs. Keeping these parameters in consideration, the optimum concentration of CS, vol. of sTPP, concentration of AA, pH of CS solution and ratio of CS/sTPP was 0.1%, 1 mL, 2%, 4.6 and 6:1, respectively, which in fact implies to test sample no. 5. So, test sample no.5 is further studied for the evaluation of NPs prepared using β-CD and SBE_7_ β-CD.

### 3.3. Characterization of Prepared NPs

#### 3.3.1. Particle Size, Polydispersity Index, Zeta Potential

The aim of the study was to develop and evaluate an innovative drug carrier system, particularly by a complexing CD with a hydrophobic drug and further incorporating the complex into the nanoparticulate system. The rationale behind this system is:

1. Cs/sTPP NPs are considered the best solubilization technique for insoluble drug substances.
2. Recently, CD complexed CS/sTPP NPs have been reported to have beneficial effects for hydrophobic drugs.
3. EPL has demonstrated the ability to interact with β-CD, a characteristic that could enhance the drug solubility, stability, and drug release profile, thereby increasing its ability to successfully incorporate into the NPs matrix system.

The size of NPs is yet another important aspect, as these nanoscale particles have the ability to cross the biological membrane. Hence, a reduction in particle size may lead to more efficient drug distribution. Therefore, NPs were synthesized in two-step processes. In the first step, an inclusion complexation was developed between EPL with β-CD and EPL with SBE_7_ β-CD using three different approaches (PM, KM and CP). In the next step, CS/sTPP NPs were prepared using the ionic gelation method. The process of nanoparticle development incorporates the specific interaction of CS and CD, leading to its ionic interaction with sTPP. Table 4 shows the physico-chemical properties of EPL/CD complexed CS/sTPP NPs. The results show that the size of all the formulations was in a nano-metric range with low PDI values and positive zeta potential. The sizes of NP1, NP3 and NP5 were 314.7 nm, 263.1 nm and 247.5 nm, respectively. When considering NP2, NP4 and NP6, the sizes were 272.2 nm, 262.5 nm and 241.5 nm, respectively. It can be concluded from the results of Table 4 that complexing EPL with either β-CD or SBE_7_ β-CD did not affect the sizes of the NPs substantially. The value of PDI is the key element that signifies the uniformity of the dispersion. The PDI values of all the NPs did not show any significant difference and lay in the range of 0.493–0.302 (Table 4). The PDI values of all the formulations were found to be less than 0.4, except NP1. Agglomeration of β-CD in water is the reason behind the significant increase in the size and PDI of NP1 [39]. The development of EPL/β-CD and EPL/SBE_7_ β-CD CS/sTPP NPs had an important impact on the overall zeta potential (ζ). Table 4 depicts the ζ of all the NPs. The values of all the NPs were positive and lay in the range of 22.7–38.9 mV. The positive values of the formulation are a clear indication that the charge on the surface of CS is not disguised, especially while preparing NPs from β-CD and SBE_7_ β-CD [40]. The overall positive value is required to prevent aggregation of particles and to facilitate the interaction with the biological membrane [41].

#### 3.3.2. Entrapment Efficiency (EE)

It was presumed that SBE_7_ β-CD complexation had an important contribution to the formation of NPs and benefited the features of NPs system when compared to β-CD. This assumption has been confirmed by an enhancement in entrapment efficiency (EE). EPL complexed with SBE_7_ β-CD not only effectively encapsulated into the nano-system but also had ionic interaction with negatively charged EPL/SBE_7_ β-CD complex, thereby improving the physico-chemical properties of NPs. The efficacy of EPL encapsulation is being determined as a two-step process:

1. The complexation of EPL with CDs.
2. The inclusion of the complexed EPL into the nano-carrier system.

The EE of all the prepared NPs during loading of EPL complexed CDs were conducted by employing the indirect method through UV-VIS spectrophotometer in the wavelength at 388 nm (Table 5). When comparing the results of β-CD complexed EPL, the EE of NPs showed a slight decrease. From the obtained results, it can be depicted that about 76% of the drug has been entrapped into the nano-system when complexing EPL with β-CD. On the other hand, EPL showed an increase in the entrapment of EPL when complexing with SBE_7_ β-CD; thereby, 80% drug complex has been incorporated in the NPs.

#### 3.3.3. Yield (%)

The ionic gelation method was employed for NP fabrication using chitosan and was susceptible to various process parameters. The fabrication of CS/sTPP NPs implies optimization of a variety of parameters, such as concentration of CS, the volume of sTPP, concentration of AA, the pH of CS solution and the volume ratio of CS/sTPP; on the other hand, the temperature of the system and the stirring speed was held constant. From Table 5, it can be seen that NPs prepared using EPL and β-CD have relatively low yield as compared to the NPs prepared when complexing EPL with SBE_7_ β-CD. Keeping in view the concentration of CS, 0.1% *w/v* was deemed essential for the development of NPs. Similar to the concentration of sTPP, 0.1 %*w/v* was selected for the analysis as the concentration beyond this resulted in aggregation of the particles. Similar work for the concentration of sTPP was reported by [42]. Three different ratios of CS/sTPP of 5:1, 6:1 and 7:1 were selected in our study and 6:1 was selected as the optimized ratio for the fabrication of NPs. The percentage yield of all the NPs was calculated and was found in the range of 44.28–52.85%. This improvement in the overall yield of NPs is desirable since it is favorable for low production costs.

#### 3.3.4. Fourier Transform Infrared Spectroscopy (FT-IR)

**Figure 1:**
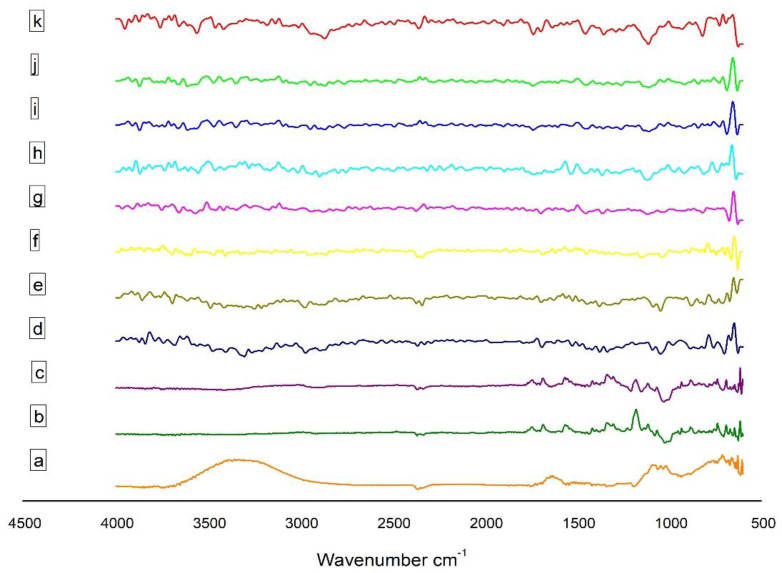
FT-IR of (**a**) EPL, (**b**) β–CD, (**c**) SBE_7_ β–CD, (**d**) CS, (**e**) sTPP, (**f**) NP1, (**g**) NP2, (**h**) NP3, (**i**) NP4, (**j**) NP5 and (**k**) NP6.

EPL, β-CD, SBE_7_ β-CD and CS drug unloaded and loaded nanoparticles prepared through ionotropic gelation method were evaluated using the FT-IR technique for the identification of distinctive absorption peaks (Figure 1). For pure drug (Figure 1a), the absorption peak of 1674.2 cm^−1^ was detected, which might be due to amide stretching. The presence of thiocarbonyl stretching was confirmed by the characteristic peak at 1554.6 cm^−1^. At 1683.8 cm^−1^, the absorption band was because of carboxylic acid stretching. The presence of similar peaks of EPL was reported by [3]. In a range of 3500–3000 cm^−1^, the absorption band was formed due to relation to O-H stretching, whereas the absorption peak at 1180.43 cm^−1^ was due to C-N band stretching.

The sharp peak at 3415.8 cm^−1^ in the spectrum of CS (Figure 1d) is related to the stretching of the O-H bond and intermolecular H-bonding, coupled with the N-H stretching of primary amines. The sharp peak at 1078.17 cm^−1^ and 1421.48 cm^−1^ is due to the symmetrical stretch of C-O-C and C-N stretching, respectively. The IR spectra of CS also exhibits N-H bending at 1597.01 cm^−1^ and amide I at 1656.8 cm^−1^, amide II at 1577.72 cm^−1^ and amide III at 1323.13 cm^−1^ [21]; the mode of vibration at 2881.57 cm^−1^ and 2918.27 cm^−1^ is a clear indication of C-H stretching. The presence of the CH_2_-OH functional group is confirmed by the peak originating at 1384.85 cm^−1^. In the spectra of sTPP (Figure 1e), the peak at 1126.39 cm^−1^ and 885.30 cm^−1^ is due to the presence of P = O and P-O-P functional groups, respectively. The peaks at 1541.08 cm^−1^ and 1635.59 cm^−1^ indicate that CS has been cross-linked to sTPP.

The spectra of β-CD (Figure 1b) shows at 2925.93 cm^−1^ for C-H stretching, 3317.47 cm^−1^ for O-H stretching, 1641.37 cm^−1^ for H-O-H bending, 1151.47 cm^−1^ for C-O bond present in the COOH group and 1020.31 cm^−1^ because of the C-O-C bending [43] The spectral analysis of SBE_7_ β-CD (Figure 1c) reveals that the O-H stretching vibration is due to the peak at 3425.48 cm^−1^. The vibration of the functional groups -CH and -CH_2_ occurs at 2800–3000 cm^−1^. The H-O-H stretching is observed at the absorption band of 1641.37 cm^−1^. A sharp characteristic peak at 1161.11 cm^−1^ is due to the stretching vibration of the C-O-C functional group. The presence of an intense peak at 1043.46 cm^−1^ confirms the presence of sulfoxide stretching.

The spectra of EPL-CDs-CS NPs are shown in Figure 1. The distinctive N-H and C-O bondings of EPL stretch at 1674.16 cm^−1^ and 1716.59 cm^−1^. The absorption band of the O-H stretching lies in the range of 3300–3000 cm^−1^ and the thiocarbonyl stretching in pure EPL is found in the spectra of all NPs at 1554.58 cm^−1^. In the spectrum, the C = O group of EPL with the frequency shifting depicted that the behavior of the drug remained unchanged, and no compatibility was observed when CD complexed EPL was incorporated into the nano-system. The cross-linking between CS and sTPP hardly revealed any shift in chemical interaction, thus confirming that EPL-CDs-CS NPs were successfully fabricated.

#### 3.3.5. Powder X-ray Diffractometry

**Figure 2:**
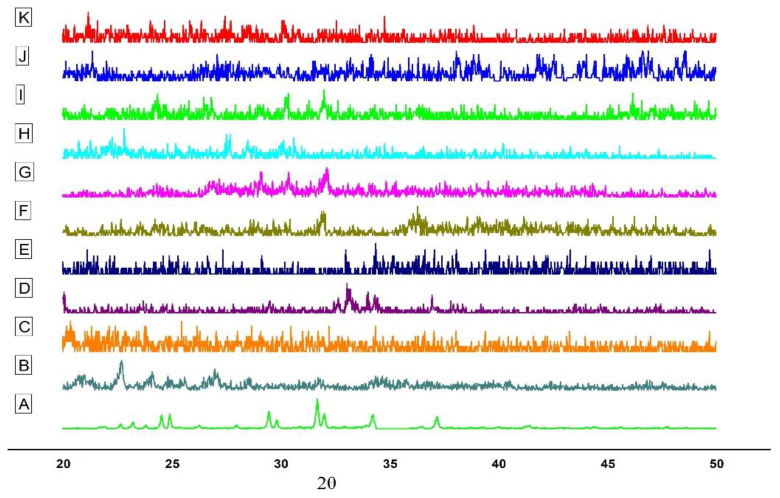
PXRD of (**A**) EPL, (**B**) CS, (**C**) sTPP, (**D**) β-CD (**E**) SBE_7_ β-CD (**F**) NP1 (**G**) NP2, (**H**) NP3, (**I**) NP4, (**J**) NP5 and (**K**) NP6.

The PXRD analysis is a popular approach to determine the crystalline nature of materials. Figure 2 illustrates the X-ray diffraction behavior of EPL, CS, sTPP, β-CD, SBE_7_ β-CD and the prepared NPs. The crystalline nature of pure EPL (Figure 2A) was revealed by XRD analysis, which revealed unique, relatively sharp, less diffuse peak values at 20–25° and 30–40°—quite similar to the peaks of EPL, as reported by Furuishi and co-workers [3]. The diffractogram of CS (Figure 2B) shows a peak at 22.7° and 26.98°, indicating the crystalline nature of CS, having a crystal lattice of 4.075 [44]. For sTPP (Figure 2C), several characteristic peaks were observed at 2θ between 20–30°, which is the clear indication for a crystalline structure foe sTPP [45]. The diffraction pattern of β-CD (Figure 2D) exhibits typical peaks prominent at 2θ of 22.6°, 22.7°, 24.1° and 27.0°. For SBE_7_ β-CD, the halo pattern with no sharp peaks, indicated the amorphous phase of CD derivative as shown in Figure 2E. For the prepared NPs, since EPL is complexed with CDs, this results in a reduction of peak intensities and shows the diffused pattern. The sharp crystal peaks of EP overlap and disappear, probably because of the hydrophilic nature of CDs that impart the wetting property resulting in improving the aqueous solubility and dissolution of the drug [31,46].

#### 3.3.6. In-Vitro Drug Release Study of EPL Loaded CS NPs

The tendency of drug molecules to be transported using a polymer to reach the active site at an adequate amount is an important factor for effective drug delivery. For this purpose, we must investigate the aspects of drug release performance and polymer degradation, while designing the nanoparticulate drug delivery systems. The rate of drug release from the NPs is affected by the following parameters:

1. Solubility
2. Drug diffusion from polymer
3. Diffusion of surface-bound and adsorbed drug
4. Erosion followed by degradation of the matrix of NPs [47]

**Figure 3:**
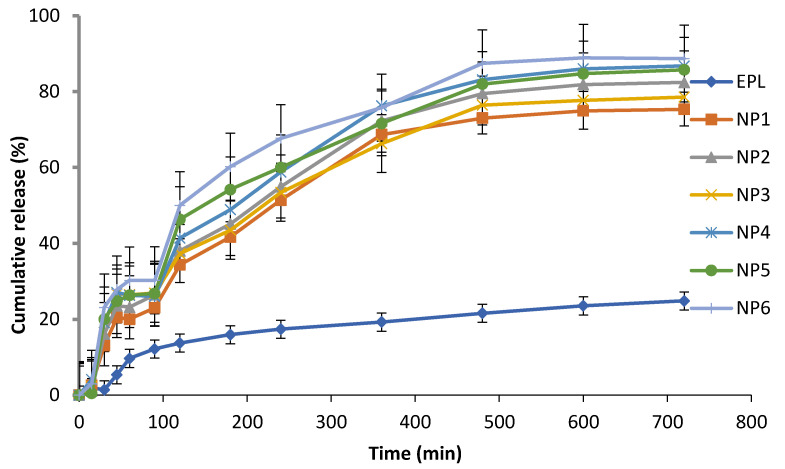
Drug release of EPL, NP1, NP2, NP3, NP4, NP5 and NP6 in a buffer of pH 6.8.

Figure 3 demonstrates the in-vitro drug release profile of EPL, CS-β-CD NPs and CS-SBE_7_ β-CD NPs using a buffer (pH 6.8). The release profile of the prepared NPs showed the burst release, followed by slow but gradual drug release. The burst release phase occurred in the first 45 min and app. 20–30% of the drug has been released. The reason for this phase might be the quick diffusion of drugs present on the surface of NPs. In the next phase, the drug is released slowly from the interior core of the NPs due to the erosion and degradation of the polymers used followed by diffusion mechanism and app. About 60–70% of the drug is released in 4–6 hrs. EPL was released at a much faster rate from EPL-SBE_7_ β-CD-CS NPs, which can be explained by the fact that SBE_7_ β-CD complexed CS NPs had a greater release effect as compared to EPL-β-CD NPs. Of all the six formulations, the release rate was highest for the formulation NP6. The cumulative release of all the NPs was arranged in ascending order as NP1 < NP2 < NP3 < NP4 < NP5 < NP6. The research study was also associated with the prior research conducted on drug-loaded CS NPs. The spontaneous drug release could be attributed to the drug-CD distribution at the surface of NPs, thus allowing fast diffusion through the porous layer of NPs [48]. The rate of drug declined after the burst phase because the release mechanism has been shifted into the diffusion phenomenon [49].

#### 3.3.7. Kinetic Modelling

The mechanism of drug release from the prepared NPs was determined using different mathematical models. The overall results of these kinetic models are crucial for selecting the most suitable formulation. The regression parameters (R^2^) derived after fitting several kinetic models for in-vitro release are given in Table 6. The values of R^2^ estimate the most suitable approach for release. The efficiency of fitness for several binary system models was valued as Korsemeyer-peppas > higuchi > 1st order > zero order. The Korsemeyer-peppas model is the best fit model. When considering diffusion exponent ‘n’, the values lie between 0.45–0.52, suggesting that the drug release from EPL-CDs-CS NPs followed the Fickian law of diffusion.

#### 3.3.8. Molecular Modelling

**Figure 4:**
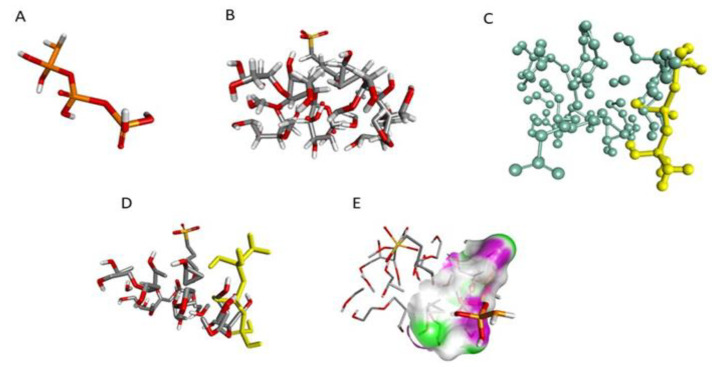
3D structure of (**A**) TPP, (**B**) SBE_7_ β-CD, (**C**) Stick-ball structure of SBE_7_ β-CD and TPP, (**D**) Docked structure of ligand and receptor, (**E**)—bonding.

For a better understanding of the relationship of cross-linker (sTPP) with SBE_7_ β-CD, in-silico molecular docking studies were performed using Biovia/Discovery studio. Figure 4 depicts the 3D structures of sTPP and SBE_7_ β-CD, and the docked structure of sTPP with SBE_7_ β-CD. The negative binding energy of −3.8 Kcal/mol suggests that TPP and SBE_7_ β-CD have been bound well and that the formation is stable. The center values of the grid box were adjusted to x= −4.671, y = −0.06 and z= −0.945. The values chosen for sizes were x = 20, y = 22 and z = 10. The gridpoint spacing of 1° A was selected for complete circling of the binding location. The docked complex was examined for binding interaction. Based on simulation studies, a part of TPP is effectively docked with SBE_7_ β-CD cavity as shown in Figure 4C. The rationale behind this is the hydroxyl group surrounding the CDs cavity Figure 4E. Thus, the CD is a narrow opening with sTPP, suggesting that this might be due to the H-bond formation [50].

#### 3.3.9. Differential Scanning Calorimetry

**Figure 5:**
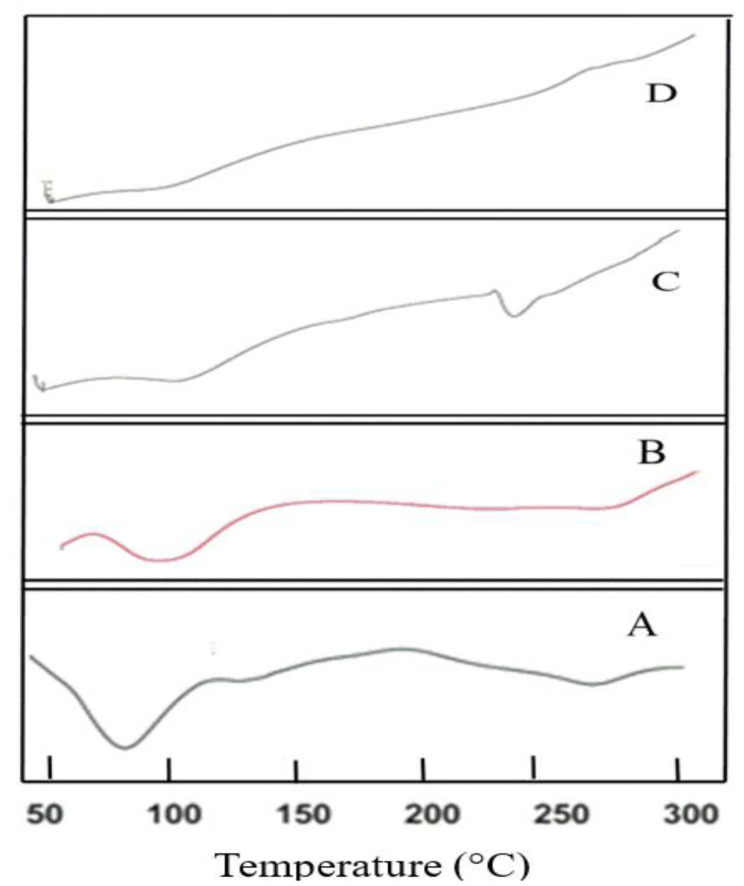
Thermal studies of (**A**) CS, (**B**) CS cross-linked with sTPP, (**C**) EPL and (**D**) NP6.

To understand the thermal behavior of the drug and the optimized formulation, DSC studies were carried out. Figure 5 shows the thermogram of CS, cross-linked CS, EPL and optimized formulation (NP6). In solid-state, polysaccharides mostly have a remarkable attraction to water, therefore, these molecules have disordered geometries that can be hydrated easily. The values of hydration for these macromolecules are chosen based on their primary and supramolecular framework. Figure 5A displays the thermogram of CS. The endothermic peak, which appeared at 93 °C, is because of the loss of water molecules. The crystalline nature of CS is altered when cross-linked with sTPP when working with pH 4.6 (Figure 5B). Cross-link CS has greater hydrophilicity, which might account for its improved water holding capacity. Therefore, a rise in polar functional groups and decrease in crystallinity attributed to an increase in water-holding capabilities of cross-linked CS. These findings are in accordance with the research work conducted by Bhumkar and Pokharkar, 2006, who examined the thermal properties of cross-linked CS at different pH values and discovered that increasing N-deacetylation and carboxymethylation resulted in improved water holding potency [51,52]. Pure EPL exhibited a sharp peak in between 200–250 °C (Figure 5C), exhibiting the crystalline nature of the EPL [3]. The thermogram of NP6 (Figure 5D) revealed an endothermic peak, which was most likely the result of the endothermic peak generated by EPL and SBE_7_ β-CD. Furthermore, the contribution of CS and SBE_7_ β-CD can be attributed to the emergence of the exothermic peak between 250–300 °C. The typical characteristic peak of EPL however, disappeared and thus cannot be observed in loaded NPs, confirming that the crystalline nature of EPL has been converted into an amorphous form.

#### 3.3.10. Morphological Evaluation

**Figure 6:**
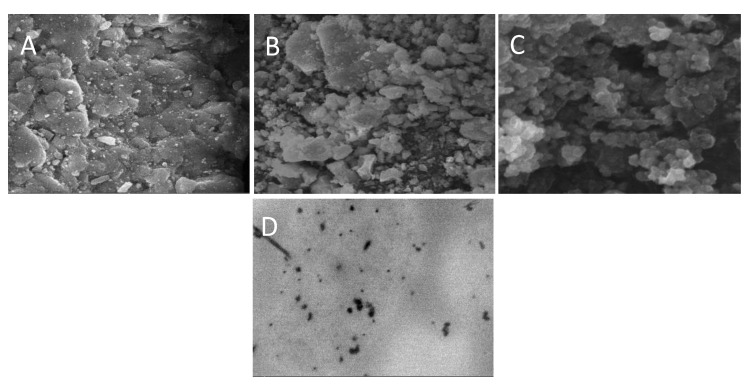
SEM images of (**A**) NP2, (**B**) NP4, (**C**) NP6 and (**D**) TEM image of EPL-SBE_7_ β-CD CS/sTPP NPs.

Figure 6 shows the SEM images of EPL loaded SBE_7_ β-CD-CS NPs prepared using the PM, KM and CP methods. The resulting images depict that drug-loaded NPs possess rough surface morphology. Figure 6A shows the SEM micrographs of NP prepared through the PM method and the images show that particles get agglomerated during the process of mixing. In comparison to Figure 6A, the images of Figure 6B,C exhibit well-defined porosity. The amorphous character of the prepared formulations with the diameter in nano-size account for the smooth surface of the NPs [53,54].

TEM analysis was used to investigate the morphology of the NPs. According to the image in Figure 6D, the NP is found to be in the nanosize range, which is similar to the results of SEM. The results are also verified by the DLS test, which confirms the NPs exact size distribution.

#### 3.3.11. Toxicity Studies 

The OECD recommendations were followed when conducting acute oral toxicity studies. The test formulation had no mortality or noticeable evidence of harmful effects when compared with Group A. With the ingestion of hazardous chemicals/test formulation, a simple and sensitive measure of harmful effects or signs of toxicity developed, resulting in either reduced body weight, shrinkage of vital organs or both. In this research study, all the measured parameters of Group A were compared to Group B and any sign of variations were analyzed. Table 7 showed the effects of the test formulation on body weight, feed, water intake, behavioral changes, and toxic effects. The test animals under observation showed a slight increase in body weight, but there was no change in behavioral patterns. The health status of all the test animals displayed no treatment-associated side effects. This implies that ingestion of test formulation has no apparent negative impact on the health of animals under observation. All animals were determined to be normal in terms of physical activities, appearances such as skin, hair, and optical characteristics. There were no symptoms of illness such as lacrimation, salvation, seizures, or hyperactivity. The animals under observation showed sensitivity and typical behavior to touch, sound, light and other thermal stimuli. Furthermore, their mobility was unrestricted, and they seemed vibrant and active. The righting and corneal reflexes were present, and the faces of the animals under study were in perfect condition with no sign of change in color and pus cells. Based on the foregoing observations and findings, it is obvious that the oral administration of test formulation had no adverse impact on the physical, growth and general behavior of test animals.

#### 3.3.12. Biochemical and Histopathological Evaluation

Blood is considered a major index to examine the pathological status in both humans and other animals. The parameters, which are routinely measured during a biochemical analysis of blood samples, included hemoglobin, pH, RBCs, TLC, eosinophil, neutrophils, monocytes, lymphocytes, PLT, MCV, MCH and MCHC. In response to any toxicity, the values of these parameters are often altered. Table 8 shows the findings of several parameters estimated in the biochemical analysis of blood. The hematological values of test animals in our study are within the reference range, which is similar to that of Group A, indicating that the developed NPs are likely to be non-toxic. To evaluate the functionality of the liver, blood levels of Alanine aminotransferase (ALT) and Aspartate transaminase (AST) were estimated. These enzymes of the liver showed no significant deviation from the normal ranges. Since the developed NPs have no hazardous impact on liver function, this proves that the test formulation is non-toxic to hepatocytes. In order to detect nephrotoxicity, the level of uric acid and creatinine were measured. Administration of test formulation did not cause any change in biochemical parameters to reach hazardous levels, therefore, they are all within the normal range.

**Figure 7:**
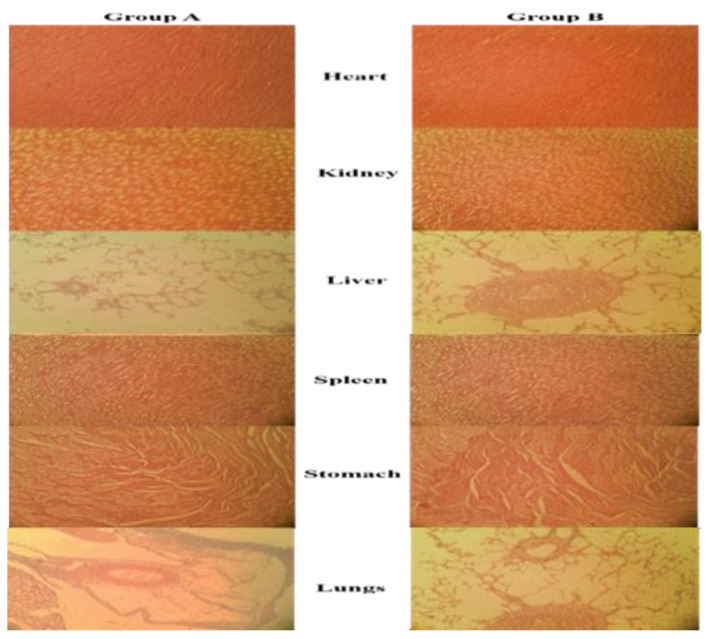
Histopathological examination of the control group (Group **A**) and treated group (Group **B**).

The histopathological analysis indicated no notable difference in the relative weight of the heart, liver, lung, kidney, and spleen of rabbits treated with test formulation in comparison to the control group as given in Table 9. These findings suggested that the NPs had no harmful effect on the organ weights of test animals. Furthermore, microscopic analysis of samples of control and treated groups revealed no obvious histopathological abnormalities on vital organs of the body. The microscopic images of cardiac muscles of the treated and control groups were compared in Figure 7. The myocardial tissues were aligned in an orderly manner and were free from inflammatory fluids, necrosis or hemorrhages. Since no substantial histological abnormalities were found in myocytes, this indicated that test formulation has no harmful effect on heart muscles. The microscopic images are of the livers of animals treated with or without test formulation. There was no apparent atrophy or necrosis as distinct lines separating each other were found between the lobules of the liver. There found no sign of hypertrophy, neutrophil, lymphocytes and macrophagic infiltration confirming no sign of toxicity in test animals. The kidney of the animal treated with test formulation was found to be normal and no sign of atrophy or hemorrhages were found in the renal glomerulus. No calcification in kidney and interstitial spaces were observed. The sinus in the spleen was found to be normal with no sign of toxicity. From all these observed parameters, it can be confirmed that the developed formulation is safe for oral administration.

## 4. Conclusions

The ionotropic gelation method was used to fabricate polymeric nanoparticles of CS encapsulating EPL-CDs. The solubility of NPs increased significantly when it was complexed with CDs. The size of the NPs was varied by changing the concentration of CS and acetic acid, volume of sTPP and ratio of CS to sTPP. In comparison to a pure drug, EPL-CDs-CS NPs improved the release of the drug. The optimization of the prepared NPs was based on the result of release studies and further evaluated for thermal behavior, surface morphology and acute oral toxicity studies. The obtained results revealed that EPL-CD-CS NPs have been shown to be safe for the oral route with no adverse effects on vital organs of rabbits.

## Figures and Tables

**Table 1:** Optimization of formulation based on changing different parameters.

Test Samples	Concentration of CS (%)	Vol. of sTPP (mL)	Concentration of AA (%)	pH of CS Soln.	Ratio of CS/sTPP
1	0.1	1	2	3.6	5:1
2	0.1	1.5	1	3.6	5:1
3	0.2	1	2	3.6	5:1
4	0.2	1.5	1	3.6	5:1
5	0.1	1	2	4.6	6:1
6	0.1	1.5	1	4.6	6:1
7	0.2	1	2	4.6	6:1
8	0.2	1.5	1	4.6	6:1
9	0.1	1	2	5.6	7:1
10	0.1	1.5	1	5.6	7:1
11	0.2	1	2	5.6	7:1
12	0.2	1.5	1	5.6	7:1

**Table 2:** Coded NPs and their description.

Coded Formulation	Description
NP1	NPs prepared by PM using β-CD
NP2	NPs prepared by PM using SBE_7_ β-CD
NP3	NPs prepared by KM using β-CD
NP4	NPs prepared by KM using SBE_7_ β-CD
NP5	NPs prepared by CP using β-CD
NP6	NPs prepared by CP using SBE_7_ β-CD

**Table 3:** Optimization of formulation based on particle size.

Test Samples	CS (%)	sTPP (mL)	AA (%)	pH of Soln.	CS/sTPP Ratio	NPs (nm)
1	0.1	1	2	3.6	5:1	510
2	0.1	1.5	1	3.6	5:1	517
3	0.2	1	2	3.6	5:1	604
4	0.2	1.5	1	3.6	5:1	781
5	0.1	1	2	4.6	6:1	314.7
6	0.1	1.5	1	4.6	6:1	358.4
7	0.2	1	2	4.6	6:1	401.7
8	0.2	1.5	1	4.6	6:1	405.7
9	0.1	1	2	5.6	7:1	861.6
10	0.1	1.5	1	5.6	7:1	905.2
11	0.2	1	2	5.6	7:1	957.6
12	0.2	1.5	1	5.6	7:1	1048

**Table 4:** Particle size (nm), polydispersity index and zeta potential (mV) of different formulations.

Formulations	Particle Size (nm)	PDI	Zeta Potential (mV)
NP1	348.4	0.578	35.4
NP2	272.2	0.366	38.9
NP3	263.1	0.493	22.7
NP4	262.5	0.302	37.3
NP5	247.5	0.327	31.8
NP6	241.5	0.363	32.9

**Table 5:** Entrapment efficiency (EE) (%) and yield (%) of different formulations.

Nanoparticles	EE (%) ± SD	Yield (%) ± SD
NP1	76.35 ± 0.002	44.28 ± 0.20
NP2	77.96 ± 0.003	47.14 ± 0.21
NP3	74.67 ± 0.002	45.71 ± 0.04
NP4	77.58 ± 0.002	50 ± 0.25
NP5	76.70 ± 0.002	48.57 ± 0.08
NP6	80.72 ± 0.003	52.85 ± 0.09

**Table 6:** Kinetic model of drug release from NPs.

Formulation	Zero Order	First Order	Higuchi	Korsmeyer-Peppas	
	R^2^	R^2^	R^2^	R^2^	n
NP1	0.7300	0.9612	0.9615	0.9630	0.528
NP2	0.7233	0.9725	0.9663	0.9671	0.521
NP3	0.6516	0.9350	0.9683	0.9691	0.481
NP4	0.6847	0.9717	0.9681	0.9843	0.499
NP5	0.6451	0.9618	0.9576	0.9582	0.484
NP6	0.5587	0.9655	0.9474	0.9533	0.451

**Table 7:** Clinical evaluation of different parameters for acute oral toxicity of Group A and Group B.

Parameters	Group A	Group B
Body Weight (kg)		
Pre-treatment	2.13 ± 0.14	2.18 ± 0.16
Day-1	2.14 ± 0.19	2.18 ± 0.22
Day-7	2.16 ± 0.27	2.19 ± 0.25
Day-14	2.17 ± 0.25	2.20 ± 0.19
Intake of Water (mL/day)		
Pre-treatment	179 ± 0.16	186 ± 0.24
Day-1	180 ± 0.17	188 ± 0.21
Day-7	183 ± 0.06	189 ± 0.33
Day-14	185 ± 0.13	191 ± 0.35
Intake of Food (g/Animal/Day)		
Pre-treatment	64 ± 0.06	65 ± 0.23
Day-1	66 ± 0.09	64 ± 0.22
Day-7	58 ± 0.17	62 ± 0.29
Day-14	63 ± 0.14	66 ± 0.21
Ocular Toxicity		
Lacrimation	Nil	Nil
Salvation	Nil	Nil
Eye irritation	Nil	Nil
Miscellaneous Effects		
Dermal toxicity	Nil	Nil
Hyperactivity	Nil	Nil
Convulsions	Nil	Nil
Touch reflexes	Present	Present
Corneal reflexes	Present	Present
Righting reflexes	Present	Present
Gripping reflexes	Present	Present
Alertness	Nil	Nil

Group A is the control group and Group B is test formulation. The values are given in mean ± SD (*n* = 3).

**Table 8:** Biochemical assessment of different parameters for acute toxicity in Group A and Group B.

Biochemical Analysis			
Hematology	Units	Group A	Group B
Hb	g/dl	14.5 ± 0.83	14.7 ± 0.94
pH		7.28 ± 0.03	7.30 ± 0.02
RBCs	10^6^/μL	6.43 ± 2.15	6.46 ± 2.25
TLC	10^3^/μL	9.1 ± 1.91	9.5 ± 2.21
Eosinophil	(%)	2.3 ± 0.13	2.4 ± 0.22
Neutrophil	(%)	48.8 ± 1.29	49.3 ± 1.33
Monocytes	(%)	2.1 ± 0.11	2.2 ± 0.13
Lymphocytes	(%)	53.8 ± 1.41	57.6 ± 1.23
PLT	10^3^/μL	334 ± 0.15	340 ± 0.13
MCV	(%)	63.4 ± 1.28	64.4 ± 1.23
MCH	pg/cell	19.4 ± 1.01	19.6 ± 0.98
MCHC	g/dl	31.0 ± 0.73	30.7 ± 0.69
**Serum Biochemistry**			
Triglycerides	mg/dl	44 ± 0.07	47 ± 0.09
Total cholesterol level	mg/dl	56 ± 0.17	62 ± 0.11
Uric acid	mg/dl	2.7 ± 0.14	2.9 ± 0.15
Creatinine	mg/dl	0.6 ± 0.03	0.7 ± 0.01
ALT	U/L	112 ± 1.26	113 ± 1.31
AST	U/L	74 ± 1.18	78 ± 1.09

Values expressed as mean ± SD, (*n* = 3).

**Table 9:** Different organ weight (g) of rabbits in Group A and Group B.

Groups	Heart	Liver	Lung	Kidney	Spleen
Group A	4.36 ± 0.08	78.24 ± 0.17	9.13 ± 0.16	12.36 ± 0.02	1.07 ± 0.04
Group B	4.18 ± 0.06	82.19 ± 0.15	9.19 ± 0.17	13.13 ± 0.04	1.13 ± 0.02

Findings expressed as mean ± SD (*n* = 3).

## Data Availability

All data available are reported in the article.
